# Text Mining and Computational Chemistry Reveal Trends in Applications of Laser Desorption/Ionization Techniques to Small Molecules

**DOI:** 10.1021/jasms.4c00293

**Published:** 2024-09-23

**Authors:** Nina P. Bergman, Jonas Bergquist, Mikael Hedeland, Magnus Palmblad

**Affiliations:** † Analytical Chemistry and Neurochemistry, Department of Chemistry−BMC, Uppsala University, SE-75124 Uppsala, Sweden; ‡ Analytical Pharmaceutical Chemistry, Department of Medicinal Chemistry−BMC, Uppsala University, SE-75123 Uppsala, Sweden; ¶ Center for Proteomics and Metabolomics, Leiden University Medical Center, 2300 RC Leiden, The Netherlands

## Abstract

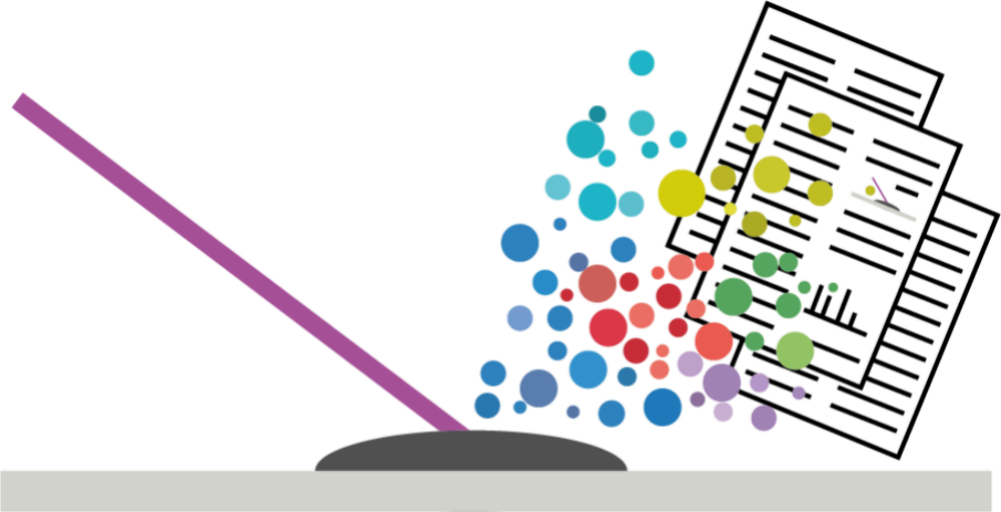

Continued development of laser desorption/ionization (LDI) since its inception in the 1960s has produced an explosion of soft ionization techniques, where ionization is assisted by the physical or chemical properties of a structure or matrix. While many of these techniques have primarily been used to ionize large biomolecules, including proteins, some have recently seen increasing applications to small molecules such as pharmaceuticals. Small molecules pose particular challenges for LDI techniques, including interference from the matrix or support in the low mass range. To investigate trends in the application of soft LDI techniques to small molecules, we combined text mining and computational chemistry, looking specifically at matrix substances, analyte properties, and the research domain. In addition to making visible the history of LDI techniques, the results may inform the choice of method and suggest new avenues of method development. All software and collected data are freely available on GitHub (https://github.com/ReinV/SCOPE), VOSviewer (https://www.vosviewer.com), and OSF (https://osf.io/zkmua/).

## Introduction

Lasers were coupled with mass spectrometry in what is now known as laser desorption ionization mass spectrometry (LDI-MS) as early as the mid-1960s.^1^ However, it was the introduction of matrix-assisted laser desorption/ionization mass spectrometry, or MALDI-MS, by Karas and Hillenkamp^2,3^ that brought the technique to the forefront, with its high efficiency and soft ionization enabling the analysis of large biomolecules. In MALDI-MS, analytes are mixed with an organic compound, the matrix, typically containing a chromophore with strong absorption in the wavelength of the laser to increase the ionization yield. While not all mechanisms or their relative importance are fully understood,^4−6^ it is well-known that some matrices work better than others for a given class of analytes, e.g. peptides,^7^ proteins,^8^ or carbohydrates.^9^ While its most frequent applications have been the analysis of such large biological molecules and synthetic polymers,^10^ MALDI has seen increased application to small molecules in recent years, including flavonoids and drugs.^11−14^ When analyzing such low molecular weight (LMW) molecules (in the mass region below 1000 Da), it is necessary to choose the matrix carefully, considering that the matrix and its fragments may overlap in the mass spectra with the analytes and their fragments, with possible saturation of the ion trap or detector in the low mass region.^15^ These issues also arise from endogenous compounds in biological sample matrices, which therefore require additional sample preparation before analysis.^11^ However, many attractive features of MALDI and other LDI techniques are independent of analyte molecular weight, including robustness, tolerance to salts and detergents, high sensitivity (given the right matrix), speed and sample throughput, ability to generate spatial information,^16^ and relative ease of use.^17^

Bibliometrics is the use of quantitative methods to describe patterns in the scientific literature to evaluate the impact of research outputs and identify trends in research topics. A bibliometric analysis of MALDI time-of-flight (TOF) MS by Li et al.^18^ revealed four major emerging trends in this field, one of which was applications to LMW analytes. This application of MALDI and other LDI techniques was reviewed in depth by Bergman et al.^19^ and Calvano et al.,^20^ who described and discussed suitable matrices for LMW analytes. In this Article, we use bibliometrics to investigate the general use of MALDI and related techniques for analyzing small molecules. As small molecules generally differ more in their physicochemical properties than proteins and other polymers, making the optimum choice of matrix more application-dependent, we specifically look at the trends in the choice of matrix for the analysis of small molecules.

Considering the large number of papers on LDI-MS techniques and their applications, literature studies and meta-analyses that limit investigations to works perceived to be the most relevant at the time of analysis may introduce bias through the selection of included publications. In this Article we take a more general approach to look at LMW applications of LDI techniques, following the method used by Palmblad et al. in the study of capillary electrophoresis.^21^ This method combines recent advances in natural language processing (NLP) and machine learning with increasing open access to publications to automatically extract information from large corpora of scientific literature without bias from making a selection of papers. We begin, however, with a more traditional overview using the VOSviewer software,^22^ which was also used recently to study of the development and applications of capillary electrophoresis.^23^

## Methods

To compare the contexts in which MALDI has been applied to the analysis of small molecules vis-à-vis peptides, proteins, and polymers, we conducted three literature searches in Europe PMC (https://europepmc.org/). For the small molecules we queried “MALDI AND (“small molecule” OR “small molecules”)”, and for the large molecules we queried “MALDI AND (peptide OR peptides OR protein OR proteins)” and “MALDI AND (oligomer OR oligomers OR polymer OR polymers)”. VOSviewer score files were generated with years of publication and number of citations for visual overlays in VOSviewer. As some records in Europe PMC have empty fields and VOSviewer does not accept missing values, we first attempted to retrieve the publication year from the “pubYear” field in the Europe PMC record. If this field was empty, we subsequently attempted to retrieve the year from the fields for the journal year of publication, first publication date, electronic publication date, and first index date, in that order. For records without any publication year, we imputed the current median publication year in all of Europe PMC (and PubMed) of 2006. For citations, we used the “citedByCount” field; when this was empty, we imputed a zero. In addition to these two scores, we divided the citation count by the age in years of the publication in a score to reveal “hot” research topics.

To investigate which matrices have been used particularly for small molecule analysis, we searched the literature for 55 known MALDI matrices with all synonyms listed in the ChEBI (Chemical Entities of Biological Interest) ontology and recorded the number of matching publications for the matrix substance by itself, the number of co-occurrences with “MALDI”, and the number of co-occurrences with “MALDI“ *and* “small molecule”, “peptide”, and “protein” (including plural forms).

To account for frequently occurring compounds that are only occasionally used as a MALDI matrix, we searched 10 common nonmatrix chemicals (adenosine, alanine, ascorbic acid, chlorophyll, cholesterol, cisplatin, cocaine, glucose, nicotine, and water), each found in over 100 000 publications, and recorded the number of co-occurrences with “MALDI”.

When generating the VOSviewer maps, we excluded uninformative terms in the titles and abstracts such as the names of months and countries. These excluded words are collected in a VOSviewer thesaurus file. When the VOSviewer files were imported from R, we normalized the threshold for the minimum number of term occurrences by the number of publications matching the query. After filtering for relevance and keeping the 60% most relevant terms, clustering was performed, and publication data overlays were computed using VOSviewer default parameters.

To look deeper into which small molecules are analyzed by MALDI-MS and related techniques, we used the SCOPE^21^ platform. Specifically, we searched all open access articles mentioning “mass spectrometry” or “MS” in their methods sections together with a specific ionization technique (Table 1) for co-occurrences with analytes matched to ChEBI entries. ChEBI entities are recognized and mined from the open access literature using NLP by Europe PMC and available through their Annotations API. Known matrix substances, defined as those compounds annotated in ChEBI as having the role “MALDI matrix material”, were removed before visualization in SCOPE, as they would be expected to coappear frequently with “MALDI” and related ionization techniques. For completeness, these queries were also analyzed with VOSviewer.

**Table 1:** Queried Ionization Techniques with Synonyms^a^

shorthand	search query
APCI^24^	(METHODS:APCI OR METHODS:“atmospheric pressure chemical ionization” OR METHODS:“atmospheric pressure chemical ionisation”)
EI^25^	(METHODS:EI OR METHODS:“electron ionization” OR METHODS:“electron ionisation” OR METHODS:“electron impact”)
ESI^26,27^	(METHODS:ESI OR METHODS:“electrospray ionization” OR METHODS:“electrospray ionisation”)
DART^28^	(METHODS:“DART” OR METHODS:“direct analysis in real time”)
DIOS^29^	(METHODS:“DIOS” OR METHODS:“desorption/ionization on silicon” OR METHODS:“desorption/ionisation on silicon”)
FAB^30^	(METHODS:FAB OR METHODS:“fast atom bombardment”)
LDI^19^	(METHODS:“LDI” OR METHODS:“laser desorption/ionization” OR METHODS:“laser desorption/ionisation”) AND NOT (METHODS:“MALDI” OR METHODS:“SALDI” OR METHODS:“SELDI” OR METHODS:“matrix-assisted” OR METHODS:“surface assisted” OR METHODS:“suface enhaced”)
NALDI^31^	(METHODS:“NALDI” OR METHODS:“nano-assisted laser desorption”)
MALDI^19^	(METHODS:MALDI OR METHODS:“matrix-assisted laser desorption/ionization” OR METHODS:“matrix-assisted laser desorption/ionisation”)
MALDI small molecules^19^	(“small molecule” OR “small molecules”) AND(METHODS:MALDI OR METHODS:“matrix-assisted laser desorption/ionization” OR METHODS:“matrix-assisted laser desorption/ionisation”)
NIMS^32^	(METHODS:“NIMS” OR METHODS:“nanostructure-initiator” OR METHODS:“nanostructure imaging”)
NIMS imaging^33^	(METHODS:“NIMS” AND METHODS:“nanostructure imaging”)
NIMS initiator^32^	(METHODS:“NIMS” AND METHODS:“nanostructure-initiator”)
SALDI^34^	(METHODS:“SALDI” OR METHODS:“surface assisted”)
SELDI^35^	(METHODS:SELDI OR METHODS:“surface-enhanced laser desorption/ionization” OR METHODS:“surface-enhanced laser desorption/ionisation”)
SIMS^36^	(METHODS:SIMS OR METHODS:“secondary ion”)
SIMS imaging^37^	(METHODS:“SIMS imaging” OR METHODS:“secondary ion imaging”)

a All queries were joined with (METHODS:“mass spectrometry” OR METHODS:“MS”) using a boolean AND. The “METHODS:” prefix restricts the searches to the methods sections of publications. In addition, the exact same queries described above for the VOSviewer analyses were also executed in SCOPE.

All Europe PMC and SCOPE queries were executed on October 1, 2023. The literature searches were orchestrated, and VOSviewer files were generated in R using the europepmc package version 0.4.1. The scripts and VOSviewer thesaurus file are available in OSF (https://osf.io/zkmua).

## Results

### VOSviewer Analyses

**Figure 1:**
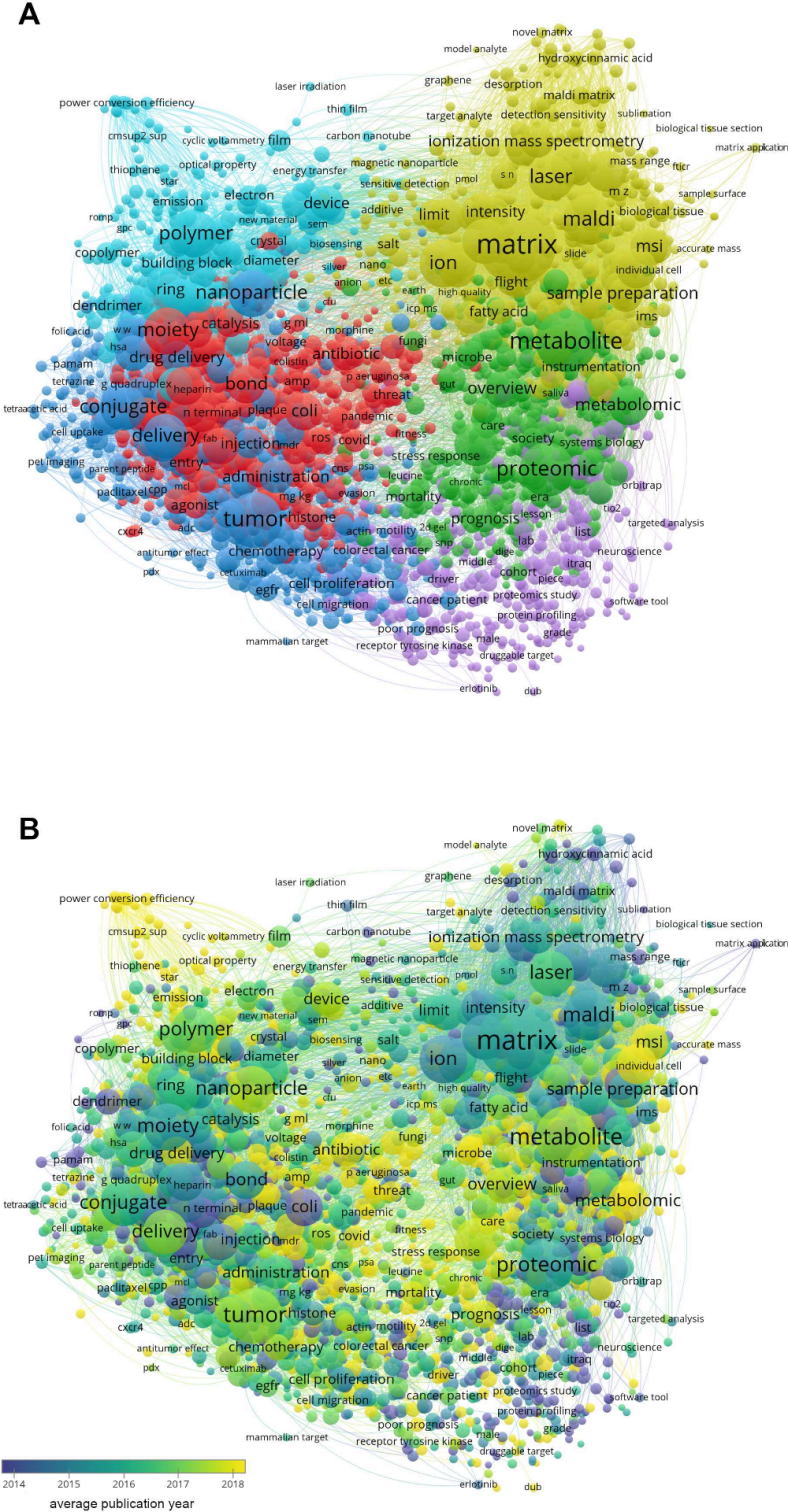
(A) VOSviewer term co-occurrence map for MALDI and small molecules and (B) average publication year overlaid on the co-occurrence map. The layout is solely based on the co-occurrences, not the publication year, citations, or any other information. A citation overlay is also available in the VOSviewer file at https://osf.io/gwx54.

**Figure 2:**
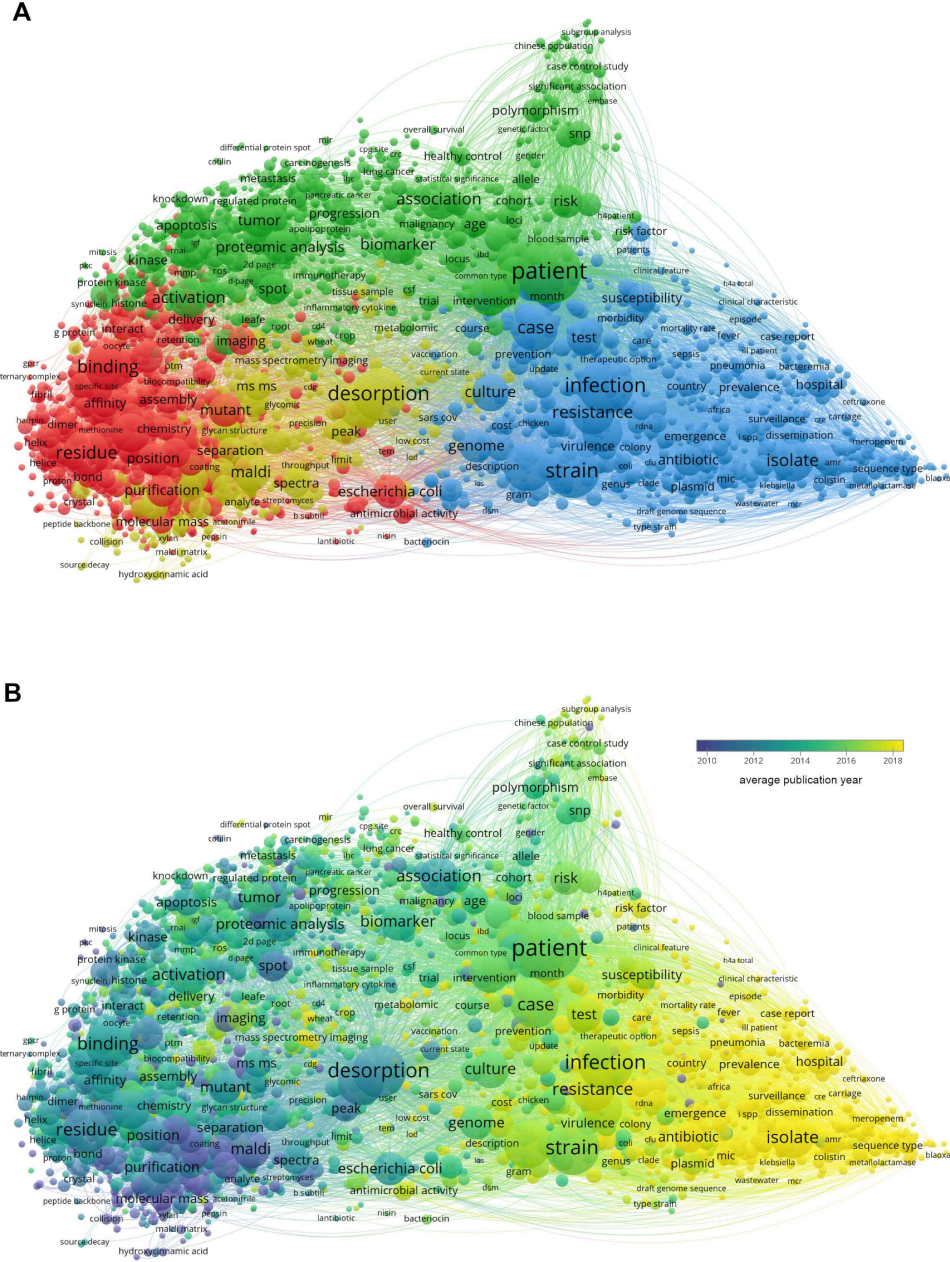
(A) Term co-occurrence map for MALDI and peptides or proteins and (B) average publication year overlaid on the co-occurrence map as in Figure 1(B). A citation overlay is also available in the VOSviewer file at https://osf.io/eyfxu.

The query for “MALDI AND (“small molecule” OR “small molecules”)” yielded 10 478 results (Figure 1), whereas the search for “MALDI and (peptide OR peptides OR protein OR proteins)” generated 80 806 results (Figure 2), with 9662 publications matching both queries. The search “MALDI AND (oligomer OR oligomers OR polymer OR polymers)” matched 23 288 publications, 4496 of which are also in the small molecule corpus. The thresholds for minimum number of occurrences were set accordingly to 10 for the small molecule(s) and 77 for the peptide(s) or protein(s), as the ratio of the number of matching publications is 80 806/10 478 ≈ 7.7.

The VOSviewer visualizations of the queries for other ionization methods are avalable on OSF (https://osf.io/zkmua) and show their corresponding applications and trends.

### MALDI Matrices

For the 10 common nonmatrix substances used to establish a baseline, 1.00 ± 0.39% of matching publications also matched “MALDI”. Out of the 55 queried matrix substances, 19 appeared together with “MALDI” in at least 5% (five times the background) of the matching publications and in at least 10 publications (Table 2). Of these 19 matrix substances, 8 are already curated as having the role of “MALDI matrix material” in ChEBI. Results for all 55 matrices are found in Supplemental Table 1.

**Table 2:** Common MALDI Matrices in the Literature Ranked by the Number of Co-Occurrences with “MALDI”^a^

matrix substance	MALDI	total	SM/PP	SM/OP
α**-cyano-4-hydroxycinnamic acid (CHCA)**^38^	9178	11431	0.16	0.53
**2,5-dihydroxybenzoic acid (DHB)**(39)	4293	7687	0.24	0.51
**trans-sinapic acid (SA)**(40)	517	4688	0.24	0.51
**3-hydroxypicolinic acid (3-HPA)**(41)	505	626	0.20	0.40
**9-aminoacridine (9-AA)**(42)	459	2183	0.38	1.05
**2′,4′,6′-trihydroxyacetophenone**(43)	421	641	0.25	0.49
1,5-diaminonaphthalene^44^	241	452	0.33	1.24
**trans-2-[3-(4-***tert***-butylphenyl)-2-methyl-2-propenylidene]malononitrile**(45)	195	199	0.35	0.22
2,5-dihydroxyacetophenone^46^	181	267	0.26	0.62
1,3-benzothiazole-2-thiol^47^	152	952	0.27	0.80
4-chloro-α-cyanocinnamic acid (CCICA)^48^	80	83	0.27	1.11
**2-(4-hydroxyphenylazo)benzoic acid**(49)	75	137	0.39	0.43
terthiophene^50^ (any)	62	837	0.82	0.38
1,8-bis(dimethylamino)naphthalene (DMAN)^51^	42	233	0.54	0.94
9,10-diphenylanthracene^52^	34	524	0.36	0.31
2,4-diphenyl-pyranylium^12^	20	24	0.58	1.22
poly(phenylenevinylene) polymer^53^	19	314	1.00	0.32
meso-tetrakis(pentafluorophenyl)porphyrin^54^	18	69	0.91	1.11
4-phenyl-α-cyanocinnamic acid amide^55^	13	13	0.58	0.88

a The numbers are the matching publications in Europe PMC queries on 2023-10-01 to the matrix substance (total), the matrix substance, and “MALDI” (MALDI); the ratio of the latter that also matches “small molecule” to those that also match “peptide” or “protein”, including plurals (SM/PP); and the ratio of those that also match “small molecule” to those that also match “oligomer” or “polymer”, including plurals (SM/OP). The higher these SM/PP and SM/OP ratios, the more preferentially the matrix is applied to small molecules. Matrices in **boldface** were curated with the role “MALDI matrix material” (CHEBI:64345) in the ontology on 2023-10-01.

The ratio between the number of matches to the matrix substance, MALDI, and small molecule(s) and the number of matches to the matrix substance, MALDI, and peptide(s) or protein(s) suggests which matrices have been particularly successful in small-molecule applications of the ionization technique. The matrices most applicable to low molecular weight analytes are meso-tetrakis(pentafluorophenyl)porphyrin, poly(phenylenevinylene) polymers, terthiophenes, 4-phenyl-α-cyanocinnamic acid amide, 2,4-diphenyl-pyranylium, and DMAN. These matrices co-occur with “small molecule” in at least half as many papers as they coappear with “peptide” or “protein” (including plural forms). The MALDI matrices most specific for peptide and protein analysis are CHCA, 3-HPA, SA, and DHB. These matrices appear more than four times as often with “peptide” or “protein” as with “small molecule” (including plurals). The ratio of the matches to “small molecule” to the matches to “peptide” or “protein” (SM/PP) and the ratio of the matches to “small molecule” to the matches to “oligomer” or “polymer” (SM/OP) are positively correlated, at least for the most frequently occurring matrices, where the SM/OP ratio is approximately twice the SM/PP ratio. The weighted average of SM/PP is 0.208, or 60% higher than the naïvely expected 0.130 from the ratio of the number of MALDI publications on peptides or proteins (80 806) to those on small molecules (10 478). Similarly for the oligomers and polymers, the weighted average of SM/OP is 0.548, compared with the expected 0.450 given the 23 288 papers matching “oligomer” or “polymer”. This suggests that publications on MALDI that explicitly mention the matrix substance (in a way that was recognized in the text mining) are more likely to also mention “small molecule” than the publications on MALDI that do not mention the matrix substance. This does not necessarily affect the comparison *between* matrices above, but it is important to consider possible bias introduced by different conventions and jargons between scientific disciplines, especially for such a broadly applied technique as MALDI.

### SCOPE Analyses

**Figure 3:**
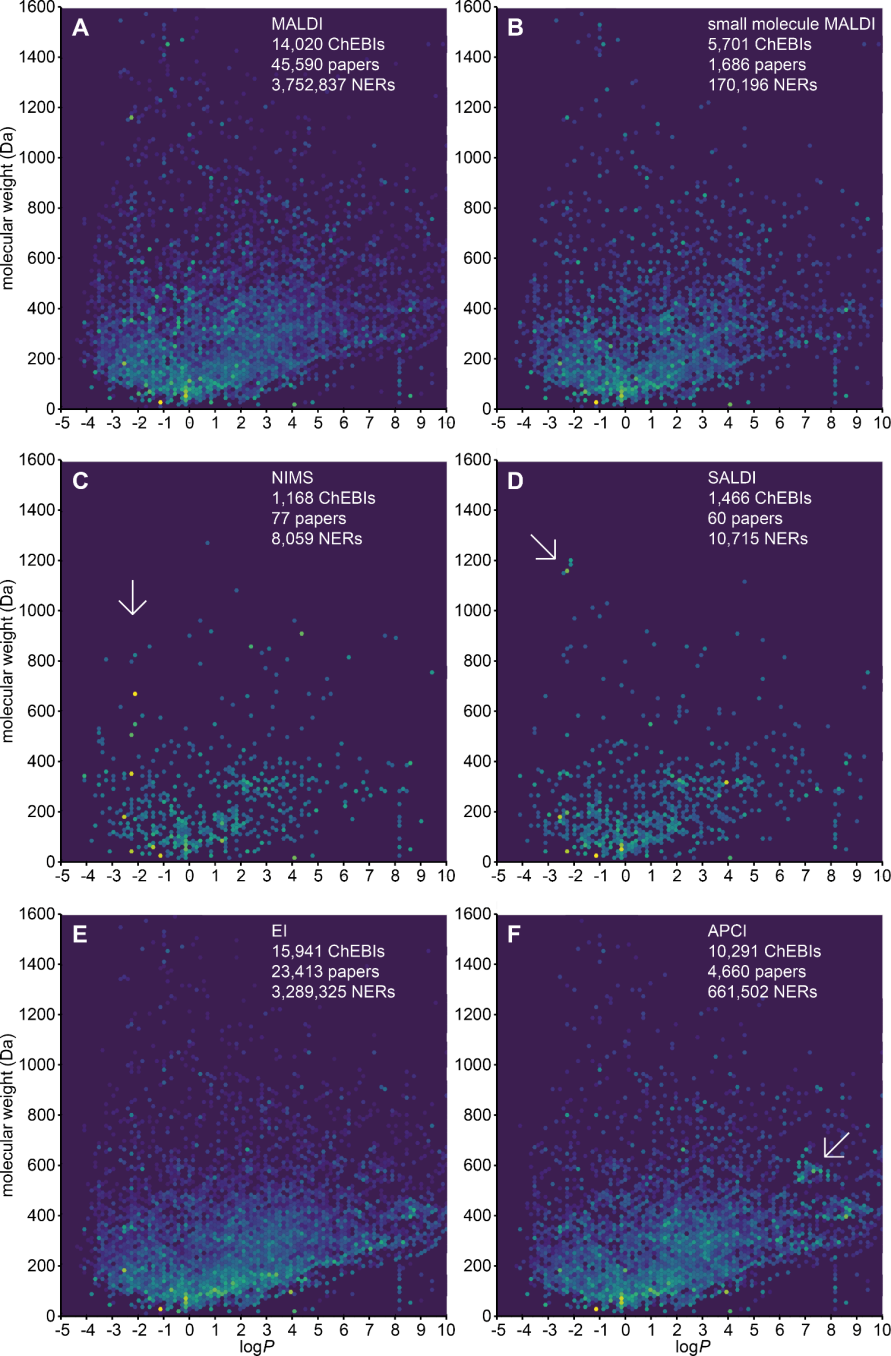
SCOPE visualization of (A) MALDI, (B) small-molecule MALDI, (C) NIMS, and (D) SALDI, with (E) EI and (F) APCI for comparison. The differences between the MALDI and small-molecule MALDI are subtle but in the expected direction, with more small molecules in the small-molecule MALDI corpus. Each panel also includes the number of unique ChEBIs annotated, the number of papers with text-mined annotations in Europe PMC, and the total number of named-entity recognitions in these papers. Oligosaccharides appear frequently in the NIMS literature with similar log *P* values around −2.3 (arrow). The SALDI literature produces a small cluster of polymyxins, colistin, and maltoheptaose (arrow). The EI results, dominated by small hydrophobic analytes such as hydrocarbons, and APCI with a “sweet spot” around 550 Da and log *P* = 7.5 (arrow) were discussed previously^56^ and are included only for comparison. An interactive version of this visualization with all queried ionization techniques and analyte classes is available at https://osf.io/rh9bs, with an unfiltered version with the matrix substances included at https://osf.io/qw3ej.

Compared with ionization techniques ESI, APCI, and EI that were previously discussed and used to demonstrate the predecessor of SCOPE,^56^ MALDI seems to have a preference, or preferred application, to large hydrophilic analytes below log *P* = 2.5 (Figure 3A). The average molecular weight of the compounds text mined in the small-molecule MALDI literature is 204.56 Da, compared to 237.90 Da for all MALDI literature. The average log *P* is 0.55 for small-molecule MALDI and 0.36 for all MALDI publications. Though the molecular weight difference is in the expected direction, one may have expected it to be larger than 33 Da. However, these corpora, as most similar literature, are dominated by very small nonanalyte compounds such as water, acetonitrile, methanol, and glucose. Larger analytes such as peptides and proteins are also generally not captured by ChEBI, except for those with given names, such as peptide hormones, neuropeptides, and antimicrobial peptides. This means that the results only partially reflect the analytes or applications of a given technique, even though the analyses do reveal general trends or differences.

The “matrix-free” NIMS technique (Figure 3C) has been used for analysis of cellulose hydrolysis products, such as cellobiose,^57^ cellotriose,^58^ xylotetraose,^59^ cellotetraose,^60^ and cellopentaose.^31^ These small oligosaccharides have similar log *P* values regardless of the molecular weight, which makes them fall on a vertical line in the SCOPE plot. The SALDI technique has indeed been used in the analysis and imaging of colistin^61^ and polymyxins.^62^ These analytes are very similar in molecular weight and log *P* and therefore form a small cluster (Figure 3D).

## Discussion

From the VOSviewer analyses, we find that terms related to method development are far more frequent in the small-molecule network (Figure 1). In the peptide and protein network (Figure 2), terms related to method development are completely overshadowed by terms associated with applications. This reflects the fact that MALDI is a mature and routinely used technique for the analysis of peptides and proteins but is still undergoing method development for small-molecule applications.

Even though peptides and proteins are not small molecules, the small-molecule network also include some of these, for example, “synuclein” and “tau”, both strongly associated with terms such as “neurofibrillary tangle”, “fibril”, “bbb”/“blood–brain barrier” and “dementia”; these are all very hot topics, with publications containing these terms having four or more citations/year (on average). “Fibril” is also connected to terms such as “amyloid”, “amyloid formation”, and “aggregation”, which have not generated as many citations, around two per year on average. Other LMW hot topics are scattered throughout the VOSviewer map, including mass spectrometry imaging (MSI) and terms related to metabolomics and microbiology, such as antibiotics, antibiotic resistance, and microbiomes.

The trend analyses in this Article combines the relative popularity of MALDI in a particular research area and the popularity of the research area as a whole, though these may be highly dependent as MALDI has enabled analyses on a (time) scale not previously achievable by other methods, such as the rapid bacterial typing of clinical isolates. In the peptides and protein network, terms related to typing, surveillance, and antibiotic resistance form a cluster (blue in Figure 2A), most of which are hot topics as measured by the average publication year. Conversely, more fundamental investigations into the MALDI process or matrices for peptides and proteins have average publication years of around or before 2010.

As is common in these types of trend analyses, some artifacts can also be seen. For example, the VOSviewer map for fast-atom bombardment or “FAB” (https://osf.io/zg8sn) contains a cluster of terms related to antibodies, revealing that many of the matching publications must have referred to the antigen-binding antibody fragment, Fab, rather than the ionization technique. We decided to keep these results in as a lesson learned and to demonstrate the usefulness of visual feedback from tools such as the VOSviewer in bibliometric analyses. Short acronyms can be ambiguous, even within a narrow domain (indeed, a few publications from the 1990s even mention both the ionization technique and the antibody fragment). We did not attempt to perform any disambiguation in these analyses.

Some matrix substances nearly always co-occur with “MALDI”, e.g., 4-chloro-α-cyanocinnamic acid and 4-phenyl-α-cyanocinnamic acid amide, suggesting this is their main or exclusive use. Also, α-cyano-4-hydroxycinnamic acid is primarily used as a MALDI matrix, with four in five publications mentioning the substance and also mentioning “MALDI”. Other compounds, such as *trans*-sinapic acid and 9-aminoacridine, also have other uses, as only 11% and 21% of publications mentioning these substances also mention “MALDI”.

These analyses reveal how different matrices have been used by practitioners in the field and suggest what works. However, it can not be ruled out that a matrix often applied to small molecules may work better than a peptide or protein matrix for some peptides or proteins or conversely that a peptide or protein matrix would not work well for some small molecules that do not overlap with matrix fragments in the spectrum, which is a common occurrence of the classical matrices often used for peptides and proteins. Matrix–analyte combinations that work are far more likely to be reported or mentioned in methods sections than combinations that do not.

Sinapinic acid shows up strongly in the SCOPE visualization of the SELDI search. A closer inspection of some of the papers seems to indicate that this might be due to the use of ProteinChiptechnology. For instance, BioRad Q10, IMAC30, CM10, NP20, and HT50 all use sinapinic acid in the recommended procedure described in the manuals for the usage of these ProteinChips.

In MALDI and other LDI techniques used for bacterial identification, we note many common antibiotics in the SCOPE visualization. Upon closer inspection, most of these papers refer to the identification of molecular signatures of strains *resistant* to the antibiotic rather than the antibiotic itself (which can even be enzymatically degraded by these strains and therefore not detectable by any ionization method). More generally, the antibiotic is outside the observed mass range when looking at ribosomal proteins^63^ or a narrow range of lipids^64^ for typing bacterial strains. For a recent review of the classes of molecules that have been used to discriminate resistant and susceptible strains, see Janiszewska et al.^65^

There is no clear difference between those substances annotated in ChEBI as having the role of “MALDI matrix substance” and those that are not (Table 2) in terms of either the number or share of publications matching both the matrix substance and the acronym “MALDI”. We have therefore submitted a suggestion to the ChEBI ontology that this role be added to these compounds and that the matrix substances not in the ontology should be added with this role. These changes to the ontology are not immediately exposed by the Europe PMC Annotations API, as the text mining is run less frequently than the monthly ChEBI updates.

### Limitations

Some acronyms, such as matrix FAB and NIMS, are ambiguous, and others, such as SEND for surface-enhanced neat desorption, also match the common English word. Even when combined with the search term “mass spectrometry”, a majority of hits match the common word. This is a familiar problem to anyone who has attempted to mine the literature for names of genes or proteins, where those with single-letter names, names matching frequently used chemicals such as SDS, or names matching words such as APP are often omitted from the results. Even with an NLP method that is 99.9% accurate in the disambiguation of these words, this remains a problem for very common words that easily outnumber the rare word by a thousand to one. The term “matrix” is uniquitous in most of the searches and is connected to most of the network. This may in part be due to homonyms, e.g., from “sample matrix”, “extracellular matrix”, or simply referring to a data matrix.

The SCOPE visualizations have a common background, and it takes some practice to spot differences and minor trends or patterns. We are working on a method to suppress background that also takes general research trends into account. However, at present, SCOPE is the only tool of its type that generates interactive visualizations of molecular properties directly from corpora of scientific literature. There may also be some bias introduced by the small numbers of research groups using NIMS or SALDI, and both techniques likely have far broader applicability than those demonstrated in the publications from these research groups.

An alternative method to estimate whether a substance is used as a matrix for small or large molecules (peptides and proteins) would have been to count the number of CHEBI annotations below a defined molecular weight and the number of occurrences of gene/protein names. However, there is no universally accepted definition of what is an LMW or “small” molecule, nor does the occurrence of gene or protein names (which are often indistinguishable) imply that these were identified or analyzed by the (MALDI) method described in the paper. It is common in fields such as metabolomics to contextualize small-molecule data using metabolic pathways and genetic or gene expression data, all containing the names of genes/proteins. All of these caveats should be kept in mind when interpreting bibliometric analyses such as the ones presented here, especially when the number of matching papers is small (less than 100 publications).

All results from queries 2023-10-01 are available at https://osf.io/zkmua/, including all VOSviewer files, SCOPE results, and metadata.

## Conclusions

Literature search and text mining can be coupled with network analysis (VOSviewer) and computational chemistry (SCOPE) to reveal or highlight trends in variants and applications of a particular analytical technique. In this work, we focused on MALDI and similar methods and compared their application to small and large molecules (peptides, proteins, and polymers).

Systematic analysis of association between MALDI matrix substances and application (analyte class) is consistent with expectation but helped fill in gaps for the less frequently used matrices. Similarly for the historical analysis, the major trends from technology development to chemical analysis and biomedical application are consistent with expectation. However, the analyses simultaneously revealed many details on specific lines of investigations. Small molecule applications are strongly trending, in particular, in contexts of mass spectrometry imaging and metabolomics.

Limitations and biases exist, such as a few matrix substances being used by a very small number of research groups with particular research interests. The correlation between these matrices and particular analyte classes may not have a direct casual relationship but is primarily influenced by the needs and choices of those groups. Ambiguous acronyms are also a challenge with current text mining methodology, although disambiguating these may be feasible with recent developments of artificial intelligence and large language models to the point where this is possible even for words like “SEND”, which very rarely refer to the LDI-MS technique.

## Supporting Information Available

Ranking of MALDI matrices in the literature (PDF)
