# Supplementary material for: Text Mining and Computational Chemistry Reveal Trends in Applications of Laser Desorption/Ionization Techniques to Small Molecules
Source: J Am Soc Mass Spectrom. 2024 Sep 23;35(10):2507–15. doi: 10.1021/jasms.4c00293 (PMC11457301; doi:10.1021/jasms.4c00293)
Supplement: Supplementary file 1 — js4c00293_si_001.pdf [file js4c00293_si_001.pdf]

Supporting Information

Text Mining and Computational Chemistry Reveal Trends in Applications of Laser Desorption/Ionization Techniques to Small Molecules

Nina P. Bergman[1], Jonas Bergquist[1], Mikael Hedeland[2] and Magnus Palmblad[3,*]

[1]Analytical Chemistry and Neurochemistry, Department of Chemistry - BMC, Uppsala University, SE-75124 Uppsala, Sweden
[2]Analytical Pharmaceutical Chemistry, Department of Medicinal Chemistry - BMC, Uppsala University, SE-75123 Uppsala, Sweden
[3]Center for Proteomics and Metabolomics, Leiden University Medical Center, 2300 RC Leiden, The Netherlands.
[*]Corresponding author email: n.m.palmblad@lumc.nl

Supplementary Table 1: MALDI matrices in the literature, ranked by the number of co-occurrences with MALDI. The numbers are the matching publications (searched with synonyms) in Europe PMC queries 2023-10-01 to the matrix substance (total), the matrix substance and MALDI (MALDI), the ratio of the latter that also match small molecule to those that also match peptide or protein, including plurals (SM/PP), and the ratio of those that also match small molecule to those that also match oligomer or polymer, including plurals (SM/OP).

matrix substance	MALDI	total	SM/PP	SM/OP
α-cyano-4-hydroxycinnamic acid	9180	11433	0.16	0.53
2,5-dihydroxybenzoic acid	4296	7690	0.24	0.51
nicotinic acid	279	30050	0.36	0.83
Z-sinapinic acid	4	29	0	0
pyrene	922	46820	0.4	0.49
quercetin	698	74490	0.39	0.64
3-hydroxypicolinic	505	626	0.2	0.4
ferulic acid	473	25077	0.27	0.41
anthracene	458	27861	0.52	0.57
9-aminoacridine	459	2185	0.38	1.05
2,4,6-trihydroxyacetophenone	421	641	0.25	0.49
anthranilic acid	231	6068	0.31	0.68
2,4-dinitrophenylhydrazine	207	5277	0.21	0.57
1,5-diaminonaphthalene	241	452	0.33	1.24
2,5-dihydroxyacetophenone	158	224	0.24	0.54
trans-2-[3-(4-tert-butylphenyl)-2-methyl-2-propenylidene]malononitrile	195	199	0.35	0.22
nifedipine	163	42808	0.48	0.78
1,2-phenylenediamine	26	775	0.1	0.15
1,3-benzothiazole-2-thiol	152	953	0.27	0.8
picolinic acid	132	3189	0.33	0.65
3-aminoquinoline	74	167	0.24	0.38
3-hydroxy-4-nitrobenzoic acid	4	30	0	NA
2,5-diaminonaphthalene	2	2	0.5	Inf
trans-sinapic acid	517	4688	0.24	0.51
4-chloro-α-cyanocinnamic acid	1	1	0	NA
2-(4-hydroxyphenylazo)benzoic acid	75	137	0.39	0.43
terthiophene	62	838	0.82	0.38
sodium ferulate	2	329	1	Inf
1,8-bis(dimethylamino)naphthalene	42	233	0.54	0.94
harmine	54	2680	0.54	0.93
tetrathiafulvalene	42	1900	0.5	0.22
9,10-diphenylanthracene	34	524	0.36	0.31
meso-tetrakis(pentafluorophenyl)porphyrin	18	69	0.91	1.11

2,4-dihydroxybenzaldehyde	16	279	0.67	0.6
4-phenyl-α-cyanocinnamic acid amide	13	13	0.58	0.88
2-(2-aminoethylamino)-5-nitropyridine	8	9	0.8	1.33
2,4-diphenyl-pyranylium	20	24	0.58	1.22
phenylenevinylene oligomers	1	23	NA	0
acenaphthene	19	2370	0.78	0.58
thiosalicylic acid	13	464	0.25	0.33
anthralin	12	1629	1.2	0.67
2,3,4,5-tetrakis(3',4'-dihydroxylphenyl)thiophene	6	6	1.25	1.67
1,8-di(piperidinyl)naphthalene	2	2	1	1
lithium vanillate	2	2	0	0
n-(1-naphthyl)ethylenediamine dinitrate	2	2	1	2
1,8-bis(tetramethylguanidino)naphthalene	5	13	0.5	0.5
2-hydrazinoquinoline	5	51	0	0
1-naphthylhydrazine hydrochloride	4	6	1.33	2
norharmane, β-carboline, 9H-pyrido[3,4-b]indole	0	0	NA	NA
4-maleicanhydridoproton sponge	2	3	Inf	Inf
2-hydrazinopyrimidine	1	9	0	NA
1,8-bis(trispyrrolidinophosphazenyl)naphthalene	1	3	NA	NA
(2E)-3-(9-anthryl)-2-cyanoacrylic acid	0	0	NA	NA
[(e)-4-(2-cyano-2-carboxyvinyl)phenyl]boronic acid	0	0	NA	NA
1,14-diaza[5]helicene	0	3	NA	NA
